# Genetic dissection of complex behaviour traits in German Shepherd dogs

**DOI:** 10.1038/s41437-019-0275-2

**Published:** 2019-10-14

**Authors:** Juliane Friedrich, Erling Strandberg, Per Arvelius, E. Sánchez-Molano, Ricardo Pong-Wong, John M. Hickey, Marie J. Haskell, Pamela Wiener

**Affiliations:** 1 Division of Genetics and Genomics, The Roslin Institute and Royal (Dick) School of Veterinary Studies, University of Edinburgh, Midlothian, EH25 9RG UK; 2 Department of Animal Breeding and Genetics, Swedish University of Agricultural Sciences, PO Box 7023, 750 07 Uppsala, Sweden; 3 Swedish Armed Forces Dog Training Centre, PO Box 194, 195 24 Märsta, Sweden; 4 Animal and Veterinary Sciences Group, Scotland’s Rural College, Edinburgh, EH25 9RG UK

**Keywords:** Behavioural genetics, Genome-wide association studies, Quantitative trait

## Abstract

A favourable genetic structure and diversity of behavioural features highlights the potential of dogs for studying the genetic architecture of behaviour traits. However, behaviours are complex traits, which have been shown to be influenced by numerous genetic and non-genetic factors, complicating their analysis. In this study, the genetic contribution to behaviour variation in German Shepherd dogs (GSDs) was analysed using genomic approaches. GSDs were phenotyped for behaviour traits using the established Canine Behavioural Assessment and Research Questionnaire (C-BARQ). Genome-wide association study (GWAS) and regional heritability mapping (RHM) approaches were employed to identify associations between behaviour traits and genetic variants, while accounting for relevant non-genetic factors. By combining these complementary methods we endeavoured to increase the power to detect loci with small effects. Several behavioural traits exhibited moderate heritabilities, with the highest identified for Human-directed playfulness, a trait characterised by positive interactions with humans. We identified several genomic regions associated with one or more of the analysed behaviour traits. Some candidate genes located in these regions were previously linked to behavioural disorders in humans, suggesting a new context for their influence on behaviour characteristics. Overall, the results support dogs as a valuable resource to dissect the genetic architecture of behaviour traits and also highlight the value of focusing on a single breed in order to control for background genetic effects and thus avoid limitations of between-breed analyses.

## Introduction

The dog (*Canis familiaris*) is a useful animal model for identifying the genetic basis of various phenotypes (Boyko 2011; Schoenebeck and Ostrander 2014) due to its favourable genetic structure, characterised by a high linkage disequilibrium and shared haplotypes across breeds (Karlsson et al. 2007; reviewed in Hall and Wynne 2012). Behavioural traits of dogs have also been shown to have a genetic component, supported by significant within-breed genetic variance (Ilska et al. 2017), pronounced differences in behavioural characteristics between dog breeds (Mehrkam and Wynne 2014; Eken Asp et al. 2015) and Belyaev’s famous “Farmed Fox” experiment in which silver foxes (close relatives of dogs) were successfully selected over several generations for increased and decreased tameness (Kukekova et al. 2012). Thus, the dog may also be a useful model for characterising the genetic architecture of behaviour and has already been used to gain insights into the genetic mechanisms underlying conditions that are also relevant in humans, such as obsessive-compulsive disorder (Dodman et al. 2010; Tang et al. 2014). In addition to such disorders, dogs may provide unique insights into the genetic basis of complex and general behaviour characteristics, including personality traits (Hall and Wynne 2012).

There are also practical concerns for studying the genetic contribution to behaviour variation in dogs. As the first domesticated species, dogs are still employed in many roles such as herding, hunting, military and police work and serving as guide dogs, but foremost, the special social bond that developed between humans and dogs has led to the dog’s popularity as a companion animal. Although dogs show tameness and strong attachment to humans in contrast to their wild ancestors, unwanted behaviours (e.g., excessive aggression, separation anxiety) still occur that affect the welfare of dogs, owners and the public (Rooney and Bradshaw 2014; Casey et al. 2014; Roth et al. 2016). Numerous studies have been performed with the aim of identifying non-genetic risk factors for the occurrence of unwanted behaviours, such as living conditions and demographic factors (Haverbeke et al. 2008; Blackwell et al. 2008; Rooney and Cowan 2011; McGreevy et al. 2013; Deldalle and Gaunet 2014; Tiira and Lohi 2015; Serpell and Duffy 2016), but few studies have considered the role of genetic factors in the management of problem behaviours. A better understanding of the genetic basis of dog behaviour may also inform breeding programmes for working dogs, e.g., guide dogs (Goddard and Beilharz 1982).

This study aims to gain general insights into the genetic architecture of behaviour variation using German Shepherd dogs (GSDs). The GSDs in this study represent unique samples of pet dogs from the United Kingdom (UK) and from a breeding programme of the Swedish Armed Forces (SAF) specifically selected for behaviour traits. By focusing on a single breed and controlling for background genetic structure that might be a consequence of analysing two populations, while also accounting for relevant environmental factors, the limitations of between-breed analyses and confounding with non-genetic effects were minimised. Moreover, different genetic approaches were applied to explore the complex nature of behaviour traits. In addition to employing a genome-wide association study (GWAS) approach based on single SNPs, a regional heritability mapping (RHM) approach was also conducted, which has been shown to perform better in the identification of multiple quantitative trait loci (QTL) with small effects (Nagamine et al. 2012). Our results highlight the complex and polygenic nature of behaviour traits and we also demonstrate that the dog is a valuable resource to study the genetic architecture of behaviour.

## Materials and methods

### Samples and phenotypes

Data on GSD behaviour and management was assessed using the Canine Behaviour and Research Questionnaire (C-BARQ) (Hsu and Serpell 2003) and a lifestyle survey (Friedrich et al. 2018). The C-BARQ consists of 101 questions related to training and obedience, aggression, fear and anxiety, separation-related behaviour, excitability, attachment and attention seeking and miscellaneous behaviours. The original C-BARQ was extended by 15 questions that assess the dog’s playfulness (Svartberg 2005; Arvelius et al. 2014) and 21 of the miscellaneous C-BARQ questions were removed due to a lack of variability (Arvelius et al. 2014), leading to 95 final questions.

The lifestyle survey consists of questions concerning demographic factors of the dog (e.g., sex, neuter status, age), its living situation (number of children, adults and other animals living with the dog, where the dog is housed) and its current and past management (puppy socialisation, exercise and stimulation, training, activities).

Owners of registered UK GSDs that were at least 2 years-old were invited to participate in the study via email by the UK Kennel Club (KC). Participating GSDs from the UK cohort were primarily pet dogs. All GSDs from the Swedish cohort were bred within the breeding programme of the SAF. After a behaviour test at the age of 15–18 months, dogs started training for working with the SAF, Swedish Police, or other authorities or companies, and/or were selected as breeding animals, whereas others were kept as companions (Wilsson and Sinn 2012). For the Swedish cohort, owners, trainers or handlers of GSDs bred within the breeding programme of the SAF that were at least 2 years-old were invited via email or letter to participate in the study.

Behaviour data and demographic and management factors were available for 1041 GSDs from the UK and Sweden (UK = 426, Sweden = 615). To calculate the behaviour traits, a principal component analysis (PCA) was applied to the data to condense the 95 questions to a smaller number of components (described in Friedrich et al. 2018). Briefly, several procedures (Cattell’s scree-test, Horn’s Parallel test and the Very Simple Structure (VSS) criterion) were applied and implemented using the R package “psych” to identify the optimal number of components that capture the important information (Abdi and Williams 2010), which gave a value of 15 for all tests. The PCA was then run for 15 principal components, followed by a varimax (orthogonal) rotation (for more information see Abdi and Williams 2010). Missing values in the data set were replaced by the median value. The dogs’ scores for the 15 components were considered as quantitative behaviour traits in the subsequent analyses.

These 15 traits describe fearful, aggressive and playful behaviours in response to humans or dogs, separation anxiety, attachment and excitability, chasing, touch-sensitivity and obedience (Friedrich et al. 2018). After correcting for fixed effects (see below), the distribution of residuals for two behavioural traits, Aversion of being stepped over and Resource guarding were significantly skewed due to dogs with extreme values. A Shapiro–Wilk test of normality revealed the highest deviations from a normal distribution for the residuals of these traits and, therefore, these traits were not considered for the following analyses, leaving 13 traits for further analysis. An overview of the 13 behaviour traits (principal components) used in the subsequent analyses is given in the supplement (S1 Table).

### Determination of non-genetic effects

Demographic and management factors were assessed with the lifestyle survey as described previously (Friedrich et al. 2018). Briefly, 28 factors were fitted in an initial linear model for each behaviour trait. Backward elimination was then applied to identify the model with the lowest Akaike information criterion (final model). These behaviour-specific final models were used in the subsequent analyses (S2 Table).

### Genotyping and quality control

DNA was extracted for 768 dogs from saliva samples collected with Performagene PG-100 swabs (UK cohort) or blood samples (Swedish cohort) using standard protocols. The genotyping was performed using the Illumina CanineHD Whole-Genome Genotyping BeadChip featuring 172,115 SNPs. When a filter for a sample call rate of > 90% was applied, 745 dogs passed the genotyping quality control. The data set was then checked using sex and relationship information estimated from the genotype data to identify potential sampling errors and four further samples were removed. The final data set included 741 dogs (UK = 324, Sweden = 417) with sex ratios of 0.8 and 0.7 (# males: # females) for UK and Swedish dogs, respectively. SNPs were filtered in GenomeStudio software (Illumina Inc., San Diego) for call rate > 98%, reproducibility (GTS) > 0.6 and signal intensity, characterised by AB R mean (mean normalised intensity of the AB cluster) > 0.3. Using PLINK version 1.9 (Chang et al. 2015), SNPs were also filtered for minor allele frequency (MAF) > 0.05 and lack of evidence for deviations from Hardy–Weinberg equilibrium (Bonferroni-corrected *P*-value of 0.05 = 4.5 × 10^−7^). Owing to allelic imbalance that can cause bias in association studies (discussed in Wise et al. 2013), SNPs on the X chromosome were removed. The final set included 78,088 autosomal SNPs.

### Pedigree and population structure

Although the GSDs in this study were from two different countries, there were shared pedigree links. Thus, the UK and Swedish pedigrees were merged into a joint pedigree, including both cohorts. To identify underlying population structure in the genomic data, a PCA was performed. To account for linkage disequilibrium between SNPs, a pruned SNP data set was used as input for the PCA, as recommended by PLINK version 1.9 (Chang et al. 2015). Genotype pruning on the filtered data set (78,088 SNPs) was performed using PLINK version 1.9 (Chang et al. 2015) based on the variance inflation factor, a function of the multiple correlation coefficient of a given SNP regressed on all other SNPs within a window (using default parameters: window size = 50 SNPs, the number of SNPs to shift the window at each step = 5, the variance inflation factor threshold = 2), leaving 9,180 SNPs as input for the PCA. The PCA was subsequently carried out in PLINK version 1.9 (Chang et al. 2015).

### Estimation of heritability

The heritability (*h*^2^) was estimated using pedigree and genotype data (the filtered data set of 78,088 SNPs). For the pedigree-based estimates, all GSDs with behaviour records (*n* = 1041) were used and the joint pedigree for the phenotyped dogs comprises 24,284 dogs. Heritability was estimated in ASReml (Gilmour et al. 2009) and GCTA (Yang et al. 2011) for pedigree-and genotype-based approaches, respectively, by fitting the following model:

1 $$y = 1\mu + Xb + Za + \varepsilon$$

where *y* is a vector of behaviour traits, *μ* is the overall mean, *b* is a vector of fixed effects with *X* as the corresponding incidence matrix, *Z* is the incidence matrix for the random additive polygenic effect, *a* is a vector of random additive polygenic effects distributed as ${\mathrm{MVN}}(0,\sigma _a^2A)$ and ${\mathrm{MVN}}\left( {0,\sigma _a^2G} \right)$ for the pedigree-and genotype-based estimates, respectively, where *A* is the pedigree-based relationship matrix and *G* is the genomic relationship matrix. *ε* is a vector of residual errors distributed as ${\mathrm{MVN}}(0,\sigma _e^2I)$, where *I* is an identity matrix. The fixed effects include the demographic and management factors that were detected to best predict the behaviour trait (S2 Table). Dogs for which one or more fixed effects were missing were removed from the analysis, such that the number of GSDs included in the analysis varied across behaviour traits (range of 906 to 1,038 and 638 to 729 for pedigree-based and genotype-based estimations, respectively) (Table 1).

**Table 1:** Heritability estimates and standard deviations for behaviour traits using pedigree and genotype data

Behaviour trait^a^	Pedigree-based^b^	Genomic-based^b^
SDA: Stranger-directed aggression (12%)	0.00 ± 0.00 (1033)	0.00 ± 0.06 (729)
DDA: Dog-directed aggression (10%)	0.00 ± 0.05 (906)	0.00 ± 0.05 (638)
SDF: Stranger-directed fear (8%)	0.04 ± 0.05 (1018)	0.04 ± 0.05 (705)
Play: Human-directed playfulness (7%)	**0.23** **±** **0.08** (1031)	**0.17** **±** **0.07** (712)
EX: Excitability (7%)	0.05 ± 0.05 (1038)	0.06 ± 0.05 (725)
SA: Separation anxiety (7%)	0.00 ± 0.00 (1010)	0.00 ± 0.05 (716)
LO: Lack of obedience (7%)	0.00 ± 0.00 (1011)	0.00 ± 0.06 (711)
SDI: Stranger-directed interest (6%)	**0.10** **±** **0.06** (985)	0.01 ± 0.05 (687)
AS: Attachment/ Attention seeking (6%)	0.00 ± 0.00 (1003)	0.02 ± 0.05 (706)
CH: Chasing (6%)	0.09 ± 0.06 (966)	**0.13** **±** **0.06** (659)
NSF: Non-social fear (6%)	**0.12** **±** **0.06** (1025)	**0.16** **±** **0.06** (727)
DDF: Dog-directed fear (5%)	0.01 ± 0.04 (1001)	0.00 ± 0.04 (698)
TS: Touch-sensitivity (4%)	0.02 ± 0.04 (966)	0.00 ± 0.04 (672)

Values highlighted in bold are significant according to a log-likelihood ratio test (for pedigree-based estimations) or for *P* < 0.05 (for genomic-based estimations)

^a^Behaviour traits are listed in descending order for the variance explained by the PCA used to define the traits (given in parentheses; see Supplement 1)

^b^Heritability estimates with the number of dogs in the analysis (given in parentheses)

The significance of pedigree-based *h*^2^ was tested using a log-likelihood ratio test (LRT) in ASReml (Gilmour et al. 2009), comparing the log-likelihood ratio statistic to a *χ*^2^ (d.f. = 1) for *P* < 0.05. The significance of genotype-based estimates was defined by *P*-values < 0.05 from the LRT within the genome-based restricted maximum likelihood (GREML) analysis performed in GCTA (Yang et al. 2011).

### Genome-wide association study (GWAS)

A GWAS was performed on the filtered data set of 78,088 SNPs to identify associations between SNPs and behaviour traits based on an additive model. To account for population structure, models with different combinations of factors (cohort as fixed effect, genotype-derived principal components 1 and 2 as covariates, genomic relationship matrix as random effect) were evaluated. Fitting only the cohort and the relationship matrix performed best, as assessed by the genomic inflation factor (*λ*) (i.e., closest to 1.0). The following linear model was fitted in GEMMA (Zhou and Stephens 2012):

2 $$y = 1\mu + Xb + c\beta + Za + \varepsilon$$

where *y* is a vector of behaviour traits, *μ* is the overall mean, *b* is a vector of fixed effects with *X* as the corresponding incidence matrix, *c* is a vector of marker genotypes (alleles coded as 0/1) with *β* as the vector of regression coefficients of the phenotype on the marker genotypes, *Z* is the incidence matrix for the random additive polygenic effect, *a* is a vector of random additive polygenic effects with ${\mathrm{MVN}}(0,\sigma _a^2G)$, where *G* is the genomic relationship matrix, and *ε* is a vector of residual errors with ${\mathrm{MVN}}(0,\sigma _e^2I)$, where *I* is an identity matrix. The fixed effects comprise the demographic and management factors obtained in the individual final models (S2 Table).

A conservative Bonferroni correction was applied to determine genome-wide significance ($P \;<\; \frac{{0.05}}{{78\,088}}$; 6.4E-07) and suggestive (allowing one false-positive per genome scan: $P \;<\; \frac{1}{{78\,088}}$; 1.3E-05) (Riggio et al. 2013) thresholds that account for the multiple testing resulting from the large number of markers but not for multiple behaviour traits.

### Regional heritability mapping (RHM)

Genomic regions were also tested for association with behaviour traits. This was carried out by scanning windows across the whole-genome using RHM, performed in REACTA (Grey et al. 2012). This approach used the model described by Nagamine et al. (2012) where two genetic effects are fitted: the first representing the overall genetic effects (modelled with an overall genomic relationship matrix calculated using all SNPs across the genome) and the second genetic effect representing the effect associated with the specific region of the genome being tested (modelled with a regional genomic relationship matrix calculated using only SNPs from this region). The SNPs used for the regional relationship matrix were excluded from the overall genomic relationship matrix (Cebamanos et al. 2014). REACTA (Grey et al. 2012) uses a sliding-window approach and we used a fixed window size of 50 SNPs with overlaps of 25 SNPs. The window size of 50 SNPs was chosen as a compromise between power to detect associations and computational demands (Uemoto et al. 2013).

Using these parameters resulted in 3124 regions under analysis; to correct for multiple testing, a Bonferroni correction was applied to genome-wide significance ($P \;<\; \frac{{0.05}}{{3124}}$; 1.6E-05) and suggestive ($P \;<\; \frac{1}{{3124}}$; 3.1E-04) thresholds.

### Analysis of candidate genes and regions

The coordinates of identified SNPs and regions were mapped to the CanFam3.1 assembly to identify (I) genes harbouring or near identified SNPs (GWAS) and (II) genes located within identified regions (RHM). Regarding (I): to determine the size of the region around identified SNPs that should be scanned for candidate genes, the squared correlation (*r*^2^) between all pairs of SNPs within 10 Mb were calculated across the genome using PLINK version 1.9 (Chang et al. 2015). The average *r*^2^ was calculated for bins of increasing distance between SNPs to identify the distance around SNPs at which average *r*^2^ drops below 0.5. The longest bin for which average *r*^2 ^> 0.5 was 200 kb and thus this distance was chosen as the region around associated SNPs to be investigated. Regarding (II), the GWAS results, −log_10_(*P*), were plotted within the regions identified by RHM to identify positional candidate genes. The pairwise *r*^2^ was calculated between all SNPs in the region and the SNP with highest −log(*P*) value to describe the pattern of linkage for the region, using PLINK version 1.9 (Chang et al. 2015) as described above. The regional associations plots were created using an R script modified from that of Saxena et al. (2007).

All genes within the regions described above (I and II) were submitted to Enrichr (Chen et al. 2013; Kuleshov et al. 2016) to identify enriched biological processes.

## Results

### Population structure

We explored the underlying population structure in the two GSD cohorts by applying a PCA to the genomic data. The variance in the genomic data explained by the first three principal components was 2.18%, 1.68% and 1.22%, respectively, and 66.96% of the variance was explained by all components with eigenvalue > 1. Plotting the first two components of the PCA (Supplementary Fig. S3) shows population structure due to cohort by a clear separation of UK and Swedish dogs based on the first principal component. However, some GSDs overlapped between the cohorts, showing shared ancestry. In contrast to the cohort effect, there were no distinct patterns observable for eigenvectors PC1 and PC2 when considering the GSDs according to their function or coat colour.

### Heritabilities

Heritability estimates for the 13 behaviour traits were calculated using pedigree and genomic data. Moderate and significant *h*^2^ were found for Human-directed playfulness and Non-social fear using pedigree and genomic approaches, while Stranger-directed interest was only significant for pedigree-based estimates and Chasing only for genomic estimates (Table 1). The highest *h*^2^ were calculated for Human-directed playfulness using pedigree data (0.23 ± 0.08) and for Non-social fear using genotype data (0.16 ± 0.06). Non-significant heritabilities were estimated for Stranger-directed fear, Excitability, Attachment/ Attention seeking, Dog-directed fear and Touch-sensitivity using estimates from pedigree and genomic data.

### Association mapping

**Fig. 1:**
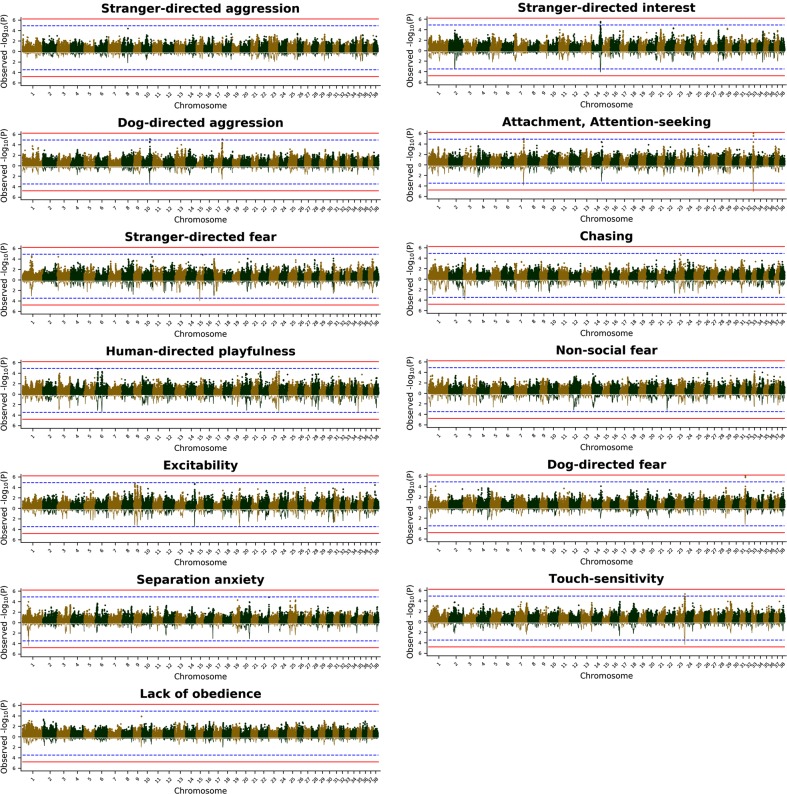
Joint Manhattan plots for GWAS and RHM analyses for the 13 analysed behaviour traits. Negative log *P*-values for each SNP and region were plotted according to their chromosomal position for the GWAS (upper plot) and the RHM (lower plot) for each behaviour trait. The red line indicates the genome-wide significance threshold and the blue dotted line indicates the suggestive threshold

Genome-wide association studies (GWAS) and a regional heritability mapping (RHM) were performed as complementary approaches to identify associations between genetic markers and the 13 behaviour traits (Fig. 1). The average genomic inflation for GWAS across the 13 behaviour traits was 0.99 (ranging from 0.89 to 1.06), showing that population stratification was adequately controlled (Supplementary Fig. S4). In the GWAS, a total of 15 SNPs were found with a suggestive association to one of the analysed behaviour traits and two of these also showed a genome-wide significant association (*P* < 6.4E-07) (Table 2).

**Table 2:** Significant and suggestive results for the genome-wide association study

Trait	Chr	Pos (bp)	SNP name	β	*p*-value	Gene(s)
AS	7	62043815	BICF2G630564528	0.27 ± 0.06	9.79E-06	*AQP4; KCTD1*
DDA	10^a^	52699559	BICF2G630490170	0.25 ± 0.06	8.06E-06	*ENSCAFG00000002716*
SDI	14	49407681	BICF2P1296430	−0.23 ± 0.05	2.78E-06	
SDI	14	49546253	BICF2S22928246	−0.23 ± 0.05	4.32E-06	
SDI	14	49864037	BICF2P325193	−0.22 ± 0.05	4.73E-06	
DDA	17^a^	59982961	BICF2P588067	−0.45 ± 0.10	9.10E-06	*ANXA9;* ***ARNT***; *BNIPL; C17H1orf56; CDC42SE1; CERS2; CTSK; CTSS; ENSA; GABPB2; GOLPH3L; HORMAD1; MINDY1; MLLT11; PRUNE1; SETDB1*
TS	23	49477874	BICF2G630364231	0.30 ± 0.07	4.82E-06	***PLCH1***; *C3orf33; SLC33A1; GMPS*
DDF	31^a^	34182127	BICF2P766705	−0.34 ± 0.07	9.17E-07	*B3GALT5;* ***BRWD1***; *HMGN1; ENSCAFG00000010048*
DDF	31^a^	34203899	BICF2P402445	−0.34 ± 0.07	9.17E-07	*B3GALT5;* ***BRWD1***; *HMGN1; ENSCAFG00000010048*
DDF	31^a^	34242284	BICF2P544489	−0.32 ± 0.07	1.66E-06	*B3GALT5; BRWD1; HMGN1; ENSCAFG00000010048*
AS	33	9217774	BICF2G630248670	0.35 ± 0.07	1.02E-06	*ENSCAFG00000009682*
AS	33	9686098	BICF2G630248946	0.41 ± 0.08	1.64E-07^b^	*ENSCAFG00000009697; ENSCAFG00000009706*
AS	33	9691453	BICF2G630248954	0.41 ± 0.08	1.64E-07^b^	*ENSCAFG00000009697; ENSCAFG00000009706*
AS	33	9705043	BICF2G630248964	0.38 ± 0.08	2.94E-06	*ENSCAFG00000009706*
AS	33	9705526	BICF2G630248967	0.38 ± 0.08	2.94E-06	*ENSCAFG00000009706*

Trait: Behaviour trait

Chr: chromosome number

SNP name: SNP ID

*β*: effect size and standard deviation

*SE* standard error or beta estimate

Gene(s): Genes located + /− 200 kb around SNP (genes harbouring the SNP are highlighted in bold)

Coordinates, statistics of the REML analysis and positional candidate genes are given for all SNPs that exceeded the suggestive or genome-wide significance threshold

^a^Locus was exclusively identified by the genome-wide association study and not by the regional heritability mapping for the same trait (all other significant SNPs were located within significant regions)

^b^Genome-wide significant association

The identified SNPs were distributed over seven of the 38 canine autosomes, with the largest numbers on CFA33 (5) for Attachment/Attention seeking, CFA31 (3) for Dog-directed fear and CFA14 (3) for Stranger-directed interest. The genome-wide associations were found for Attachment/Attention seeking (two adjacent SNPs on CFA33). The greatest number of suggestive SNPs were found for Attachment/ Attention seeking (6), Stranger-directed interest (3) and Dog-directed fear (3).

The RHM analysis was performed by testing for associations between 50-SNP sliding windows across the genome (with a 25-SNP overlap between consecutive windows) (Fig. 1). Scanning the genome for regions associated with the 13 behaviour traits based on the suggestive threshold, we identified 16 regions associated with at least one of the behaviour traits (Table 3). One region on CFA33 associated with Attachment/Attention seeking showed genome-wide significance and also harbours the only SNPs with genome-wide significance in the GWAS. The average size of the identified regions was 1.31 Mb (range: 0.89–2.63 Mb).

**Table 3:** Significant and suggestive results for the regional heritability mapping

Trait	Chr	Start (Mb)	Stop (Mb)	*h* ^2^	LRT	*P*-value	Gene(s)
SA^a^	1	33.9	34.8	0.19 ^b^± 0.09	15.5	4.05E–05	*ADGRG6; AIG1; HIVEP2*
CH^a^	3	8.72	10.3	0.03 ± 0.03	13.5	1.19E–04	*FAM174A; SLCO4C1; SLCO6A1; ST8SIA4*
CH^a^	3	9.68	11.0	0.03 ± 0.02	11.8	3.03E–04	*FAM174A; ST8SIA4*
AS^a^	7	61.4	62.5	0.03 ± 0.02	12.3	2.31E–04	*AQP4; CHST9; KCTD1; PSMA8; SS18; TAF4B*
AS	7	62.0	63.0	0.04 ± 0.02	13.3	1.33E–04	*KCTD1; PSMA8; SS18; TAF4B*
SDI	14	49.1	50.1	0.02 ± 0.01	13.6	1.16E–04	
SDI	14	49.6	50.6	0.05 ± 0.03	14.3	7.81E–05	
SDI^a^	14	50.1	51.3	0.05 ± 0.03	13.3	1.35E–04	*ENSCAFG00000003294; ENSCAFG00000003299; LRRN3*
SDF^a^	15	35.5	37.7	0.18^b^ ± 0.10	12.8	1.69E–04	*AMDHD1; CCDC38; CDK17; ELK3; ENSCAFG00000006395; ENSCAFG00000006472; ENSCAFG00000006487; ENSCAFG00000006494; HAL; LTA4H; NEDD1; NTN4*
SDF^a^	15	36.9	39.5	0.13^b^ ± 0.08	13.9	9.44E–05	*ACTR6; ANKS1B; APAF1; ENSCAFG00000006545; ENSCAFG00000006667; ENSCAFG00000006682; ENSCAFG00000023440; ENSCAFG00000006734; IKBIP; SCYL2; SLC17A8; TMPO; UHRF1BP1L*
Ex^a^	19	48.4	49.2	0.04 ± 0.03	11.7	3.06E–04	
TS	23	48.6	50.0	0.04 ± 0.03	13.4	1.26E–04	*C3orf33; GMPS; KCNAB1; MME; PLCH1; SLC33A1*
TS	23	49.3	51.0	0.04 ± 0.03	15.4	4.24E–05	*C3orf33; CCNL1; ENSCAFG00000029908; ENSCAFG00000008882; ENSCAFG00000032528; GMPS; KCNAB1; PLCH1; PTX3; SLC33A1; SSR3; TIPARP; VEPH1*
AS	33	8.18	9.57	0.05 ± 0.03	12.5	2.09E–04	*ZPLD1*
AS	33	8.85	10.1	0.07 ± 0.04	18.6	7.90E–06^c^	*ENSCAFG00000009682; ENSCAFG00000009697; ENSCAFG00000009706*
AS	33	9.60	10.6	0.07 ± 0.04	13.5	1.20E–04	*ENSCAFG00000009706; ENSCAFG00000025525*

Trait: Behaviour trait

Chr: chromosome number

Start (Mb) and Stop (Mb): beginning and end of the region

*h*^2^: estimate of heritability and standard deviation

Coordinates, statistics of the association analysis, regional heritabilities and positional candidate genes are given for all genomic regions that exceeded the suggestive or genome-wide significance threshold. Owing to the sliding-window approach used in the analysis, the regions comprise 50 SNPs and can overlap with adjacent regions by 25 SNPs

*SE* standard error of heritability, *LRT* log-likelihood ratio

^a^Locus was exclusively identified by the regional heritability mapping and harboured no significant SNP identified by the GWAS for the same trait

^b^Overestimation (regional *h*^2^ is inconsistent with the corresponding genome-wide *h*^2^)

^c^Genome-wide significant association

Most of the SNPs identified by the GWAS overlapped with regions identified by the RHM (Tables 2 and 3 and Fig. 1), only the SNPs found on CFA10 and CFA17 for Dog-directed aggression and on CFA31 for Dog-directed fear were exclusive to the GWAS approach. Exclusive peaks were also found with the RHM approach, for example on CFA1 for Separation-anxiety, on CFA3 for Chasing, and on CFA19 for Excitability.

### Candidate genes and regions

According to the annotation of CanFam3.1, four of the SNPs identified by the GWAS were located within three genes (*ARNT, PLCH1* and *BRWD1*) and 30 genes were located within 200 kb of suggestive or genome-wide significant SNPs (Table 2). The two SNPs on CFA33 with genome-wide significance for Attachment/Attention seeking were located approximately 63 kb downstream of an unannotated protein-coding gene (*ENSCAFG00000009706)*. Gene ontology analysis of the 30 genes revealed that the top enriched biological processes were “polyphosphate metabolic process” (GO: 0006797; adjusted *P*-value = 0.009), “negative regulation of axon regeneration” (GO: 0048681; adjusted *P*-value = 0.12) and “regulation of hormone biosynthetic process” (GO: 0046885; adjusted *P*-value = 0.12).

To further investigate regions identified by the RHM analysis, −log(*P*) values obtained from the GWAS, gene annotations and local linkage disequilibrium patterns were plotted for these regions to pinpoint the most likely location of positional candidate genes (Supplementary Fig. S5). Overlapping regions, due to the sliding-window approach of the RHM analysis, were combined. There were 60 genes located in these regions (Table 3); of these, several functional candidate genes (*LRRN3, KCNAB1* and *BRWD1*) were also located near (Supplementary Fig. S5) or at (Table 2) SNPs identified by GWAS. Two other functional candidate genes (*HIVEP2* and *AIG1*) were located in identified regions but the −log(*P*) values for nearby SNPs obtained in the GWAS did not exceed the suggestive threshold (Supplementary Fig. S5). The region on CFA33 with genome-wide significance for Attachment/Attention seeking comprised three unannotated protein-coding genes (*ENSCAFG00000009682*, *ENSCAFG00000009697* and *ENSCAFG00000009706*).

According to the gene ontology analysis, the GO biological processes significantly enriched by genes located in identified regions (Table 3) were “histidine catabolic process” (GO: 0006548; adjusted *P*-value = 0.013), “histidine metabolic process” (GO: 0006547; adjusted *P*-value = 0.013) and “imidazole-containing compound catabolic process” (GO: 0052805; adjusted *P*-value = 0.013).

## Discussion

Dogs express diverse behaviour phenotypes, some of which appear to be related to traits of other species (including humans), making them useful models for general insights into the genetic architecture of behaviour. However, behaviours are complex traits, which have been shown to be influenced by numerous non-genetic (environmental) factors and genetic variants of low to moderate effect (Flint 2003), which complicates their analysis and the identification of underlying genes and mechanisms. In this study, we analysed the influence of genetic factors on behaviour traits of German Shepherd dogs using multiple genomic approaches, while accounting for various non-genetic factors, with the aims of characterising the general genetic architecture of behaviour and identifying candidate genes.

### The genetic contribution to behaviour variation

The heritabilities estimated for the 13 behaviour traits using pedigree and genomic approaches ranged from 0 to 0.23. These measures for *h*^2^ are within the range of most previously observed values in dogs (Saetre et al. 2006; Arvelius et al. 2014; Ilska et al. 2017), while a few studies reported higher *h*^2^ for similar behaviour traits (Ruefenacht et al. 2002; van der Waaij et al. 2008). Discrepancies between observed *h*^2^ for dog behaviour traits across studies can be explained by the different behaviour phenotypes used, e.g., whether the behaviour was subjectively scored or actually measured and whether the behaviour was recorded in everyday life or in test situations, and also by differences between breeds (due to different population histories).

From other species it is known that specific behaviour patterns contributing to the fitness of an individual, such as courtship or feeding, are under stronger genetic control than behaviours with apparently less evolutionary relevance like personality traits (York 2018). In this study, behaviour traits with substantial *h*^2^ were Human-directed playfulness, Non-social fear, Stranger-directed interest and Chasing. The observation of the highest *h*^2^ across traits for Human-directed playfulness has been also made in a genetic study of 14 different dog breeds (Asp et al. 2014). While many other studies on the genetic background of dog behaviour focused on human-directed aggression (Liinamo et al. 2007; Våge et al. 2010; Zapata et al. 2016), we included traits of playful interactions in our analysis since playfulness in regard to humans has been shown to explain a large proportion of the variance between individuals in the analysis of multiple dog breeds (Svartberg 2005). In particular, Human-directed playfulness and Stranger-directed interest describe boldness and attachment to humans and our results indicate that these behaviour characteristics might be directly targeted by selection for tameness and human-attachment in dogs. Specifically regarding GSDs, although the SAF do not use C-BARQ for their selection programme, a previous study showed significant associations between success in a temperament test assessing dogs for further training and C-BARQ-measured traits of young dogs related to Lack of obedience, Stranger-directed fear, Non-social fear, Dog-directed fear and Touch-sensitivity (Foyer et al. 2014), suggesting that these traits have been selected against in the Swedish cohort. We do not have similar information for the UK cohort as these dogs are primarily pets and not part of a breeding programme, however, it is possible that selection criteria over recent years have been based more on cosmetic traits as the breed has moved from a working dog to pet (O’Neill et al. 2017).

Using genome-wide association and regional heritability mapping, we identified 15 SNPs and 16 regions, respectively, which showed suggestive association with one of the analysed behaviour traits. These SNPs and regions were distributed over 11 chromosomes. Several regions were identified by both GWAS and RHM.

Comparing genomic regions identified in the current study to the results from other single-breed studies, we found that the SNP for Attachment/Attention seeking on CFA7 is located in a region of ~1 Mb flanked by two loci associated with obsessive-compulsive disorder in Doberman Pinschers (Tang et al. 2014). In contrast, the suggestive SNPs identified for behaviour traits in Labrador Retrievers by Ilska et al. (2017) do not overlap with candidate regions found in the current study. Furthermore, none of the genetic regions mapped to aggression and fear across multiple dog breeds in a study by Zapata et al. (2016) overlapped with genetic regions found in the current study. Ostrander et al. (2017) reviewed the identified loci for behaviour traits across dog breeds by Zapata et al. (2016) and found that many of these loci were previously linked to body size, suggesting that behaviour may have been confounded with physical characteristics in between-breed analyses or an association between behaviour and some morphological traits. In the silver fox experiment described above, changes in behaviour were also accompanied by physiological and morphological changes (Trut 1999) and other studies have shown an association between behaviour and body traits across breeds (McGreevy et al. 2013), suggesting an genetic interplay between these traits. These observations might also indicate that GWAS across dog breeds are more sensitive for morphological differences than for variation in behaviour, which highlights the importance of single-breed analyses in the dissection of the genetic background of behaviour. In contrast to the Zapata et al. (2016) study, candidate regions identified in the current study do not overlap with known genetic regions associated with body size (based on the largest study to date, Hayward et al. 2016).

However, our results also suggest that QTL for dog behaviour may be breed-specific as indicated by the lack of QTL that overlap those found in other studies. It is likely that across breeds, different behaviour-oriented breeding practices have led to different alleles selected to moderate frequencies, leading to breed-specific QTL.

### Candidate genes related to behaviour traits

In this study, we combined two complementary approaches (GWAS and RHM) with the aim of detecting novel candidate genes for behaviour and further evaluating genes previously linked to behaviour.

The only SNPs and region with genome-wide significance for the behaviour trait Attachment/Attention seeking point to a region on CFA33 that contains several unannotated protein-coding genes, including *ENSCAFG00000009706*. According to the iDOG database (Tang et al. 2019), *ENSCAFG00000009706* is a protein-coding gene with molecular functions related to RNA binding and the structural constitution of the ribosome (GO: 0003723 and 0003735). However, this gene has not yet been described in other canine association mapping studies.

Many of the other positional candidate genes have been previously linked to behaviour characteristics and disorders or to neuronal development, especially in regards to humans. The aquaporin-4 (*AQP4*) gene identified by both GWAS and RHM for Attachment/Attention seeking is one of the most abundant molecules in the brain, with many physiological functions (reviewed in Nagelhus and Ottersen 2013). In a study on gene expression changes in the brains of dogs and wolves, *AQP4* showed a significant fourfold higher gene expression in dog than in wolf, indicating that it may have played a role in domestication (Saetre et al. 2004). Our results provide further evidence for the role of this gene regarding attachment to humans.

RHM identified several regions that were not identified by the GWAS and contain genes that have previously been linked to behaviour. The region at ~34 Mb on CFA1, associated with Separation anxiety, includes *HIVEP2* and *AIG2*, which have been previously identified as positional candidate genes in a GWAS on affiliative social behavior in humans (Knoll et al. 2018). The region at 50–52 Mb on CFA14, associated with Stranger-directed interest, includes *LRRN3*, a strong risk gene for autism in humans (Hutcheson et al. 2004). In addition, the region at ~49–51 Mb on CFA23, associated with Touch-sensitivity (a behaviour trait that is characterised by fearful or aggressive responses to grooming or bathing), contains another promising functional candidate gene, *KCNAB1*. Two SNPs with low but not quite suggestive *P*-values in the GWAS were also located within the *KCNAB1* gene, which encodes the voltage-gated potassium channel subunit beta-1. Interestingly, mouse knockouts at the *KCNQ* gene, which encodes another voltage-gated potassium channel, showed an increased sensitivity of mechanoreceptors in the skin (Schütze et al. 2016). It is possible that variation in *KCNAB1* could have a similar effect and thus this might influence touch-sensitivity in dogs.

The GO analysis for genes identified by the RHM revealed an enrichment of catabolic and metabolic histidine processes due to the genes *AMDHD1* and *HAL* (the region harbouring these two genes was associated with Stranger-directed fear). Histidine is a precursor of the neurotransmitter histamine and it has been shown that the histaminergic system affects the central nervous system and thus also alters behaviours, e.g., by affecting fear memory (reviewed in Passani et al. 2007).

Other genes were identified only by the GWAS, including *BRWD1* (CFA31)*, B3GALT5* (CFA31) and *ARNT* (CFA17). Two SNPs associated with Dog-directed fear are located within *BRWD1*. In human GWAS studies, this gene has been associated with cognitive function (Davies et al. 2018), intelligence (Savage et al. 2018) and temperament in individuals with a bipolar disorder (Greenwood et al. 2012). In close proximity to these SNPs lies *B3GALT5*, which has been linked to suicide attempts (Perlis et al. 2010) and obsessive-compulsive symptoms (den Braber et al. 2016). Finally, a SNP on CFA17 associated with Stranger-directed interest is located within the *ARNT* gene. Variation within *ARNT* has been linked to the severity of autism in humans (Fujisawa et al. 2016).

### Limitations and implications for further studies

The limited number of genome-wide significant associations found in this study indicates the challenges in the genetic dissection of complex traits like behaviour, which derive from the small effects of genetic variants on phenotypic variation, substantial environmental effects and difficulties in defining clear phenotypes. Although ours is one of the largest genomic studies of dog behaviour so far, it has been shown in human studies that much larger sample sizes are required for robust genetic dissection of complex traits, e.g., height (Visscher et al. 2014). The use of C-BARQ, a standardised owner-derived questionnaire, to measure behaviour phenotypes, which has been successfully applied in many studies and records a range of behaviours in everyday situations, opens the possibility of meta-analysis across studies and thus ultimately achieving a larger sample size. However, a limitation of using questionnaire-based phenotypes is that the recorded traits are influenced by the subjectivity of the participants, which might be even more pronounced when participants originate from different countries and thus show cultural differences as in this study. While we attempted to correct for this in the statistical analysis, we may not have been completely successful.

## Conclusions

Understanding the genetics of dog behaviour and the interaction with non-genetic factors can give general insights into animal and human behaviour and is relevant for animal welfare, e.g., to identify risk factors for problem behaviours. Our results support the hypothesis that behaviours are complex traits, influenced by multiple genetic and non-genetic factors, emphasising the need for large datasets incorporating both genetic and non-genetic information in future studies of dog behaviour. Furthermore, it is important to reach a consensus on the non-genetic factors with greatest effects on these traits in order to standardise analyses.

If these requirements are met, dogs can provide a valuable resource for studying the genetics of behaviour characteristics, especially in terms of intra- and inter-species social interactions. In this study, genomic regions and SNPs associated with behaviour traits suggested a number of candidate genes that were previously described for psychological disorders in humans, indicating a potential new context for these genes in the general expression of behaviour variation. By analysing a single dog breed, we were able to highlight candidate genes for behaviour that are less likely to be confounded with morphological variation compared to between-breed analyses. However, further studies with larger sample sizes are required to identify and confirm the identified associations and candidate genes and, where associations are confirmed, subsequent functional analyses will be needed to progress in understanding how these genes influence expression of behaviour.

### Data archiving

Data for the UK dogs is available from the Dryad Digital Repository: 10.5061/dryad.493rk16.

## Supplementary information

Supplementary material
