# Supplementary material for: Genetic dissection of complex behaviour traits in German Shepherd dogs
Source: Heredity (Edinb). 2019 Oct 14;123(6):746–58. doi: 10.1038/s41437-019-0275-2 (PMC6834583; doi:10.1038/s41437-019-0275-2)
Supplement: Supplementary file 1 — Supplementary material [file 41437_2019_275_MOESM1_ESM.docx]

**S1 Table**. Description of the behaviour traits used as phenotypes. Behaviour traits were generated using a principal component analysis (PCA) on questions from the C-BARQ questionnaire and additional questions about playfulness.

| Behaviour trait | Description |
| --- | --- |
| Stranger-directed aggression (SDA) | Positive loadings of aggressive behaviour towards strangers |
| Dog-directed aggression (DDA) | Positive loadings of aggressive behaviour and negative loadings of playfulness towards unfamiliar dogs |
| Stranger-directed fear (SDF) | Positive loadings of fearful behaviour towards strangers |
| Playfulness (Play) | Positive loadings of playful interaction with humans |
| Resource guarding (RG) | Positive loadings of owner-directed aggression in regard to food or toys |
| Excitability (EX) | Positive loadings of excited behaviour in response to different situations |
| Separation anxiety (SA) | Positive loadings of stress-related behaviour when left alone |
| Lack of obedience (LO) | Negative loadings of obedience-related behaviours |
| Stranger-directed interest (SDI) | Positive loadings of friendly interaction with strangers |
| Attention seeking (AS) | Positive loadings of attention-seeking behaviour towards owner |
| Chasing (CH) | Positive loadings of chasing-related behaviours |
| Non-social fear (NSF) | Positive loadings of fear response to loud noise or unfamiliar objects |
| Dog-directed fear (DDF) | Positive loadings of fearful behaviour towards unfamiliar dogs |
| Aversion of being stepped over (AvSO) | Positive loadings of fearful or aggressive response when stepped over |
| Touch sensitivity (TS) | Positive loadings of fearful behaviour when touched for various treatments |

**S2 Table.** Lifestyle variables that were fitted as fixed factors in the statistical analyses of behaviour traits. Description of lifestyle variables that were assessed using the lifestyle survey ( “Variables”) and individual models for every behaviour trait where variables fitted as fixed effects in the models are indicated by “x” (“Models”).

*Variables*

| **Name** | **Description** | **Type** | **Levels** |
| --- | --- | --- | --- |
| Cohort | Origin of the dog | Categorical | UK |
|  |  |  | Sweden |
| People_hh | Number of people in household | Numerical | n.a. |
| Children_hh | Number of children < 15 years in household | Numerical | n.a. |
| Dogs_hh | Are there other dogs living in the household? | Binary | Yes |
|  |  |  | No |
| Animals_hh | Are there other animals living with the dog (e.g. cats, rodents, livestock)? | Binary | Yes |
|  |  |  | No |
| Living place | Where does the dog live? | Categorical | 1: Inside |
|  |  |  | 2: Primarily outside |
|  |  |  | 3: Indoors and outside |
|  |  |  | 4: Place of work |
| Age | Age of the dog in years | Numerical | n.a. |
| Gender | Gender of the dog | Binary | Female |
|  |  |  | Male |
| Neuter status | Has the dog been neutered? | Binary | Intact |
|  |  |  | Neutered |
| Gender*Neuter status | Interaction of gender and neuter status | Categorical | 1: Female*intact |
|  |  |  | 2: Female*neutered |
|  |  |  | 3: Male*intact |
|  |  |  | 4: Male*neutered |
| Coat colour | Coat colour of the dog | Categorical | 1: Typical GSD coloured^C^ |
|  |  |  | 2: Sable |
|  |  |  | 3: Black |
|  |  |  | 4: Other |
| Age.acquisition | How old was the dog when acquired? | Ordinal | Birth |
|  |  |  | < 9 weeks |
|  |  |  | 9 - 12 weeks |
|  |  |  | 12 - 16 weeks |
|  |  |  | 4 - 6 months |
|  |  |  | 6 - 12 months |
|  |  |  | 1 - 2 years |
|  |  |  | 2 - 4 years |
|  |  |  | > 4 years |
| Bred | Has dog been used for breeding? | Binary | Yes |
|  |  |  | No |
| Shape | Shape of the dog with increasing body weight | Ordinal | A |
|  |  |  | B |
|  |  |  | C |
|  |  |  | D |
| Role_PC1 | How do you see your dog? | PCA score^P^ | Pet (0.77) |
|  |  |  | Playmate (0.78) |
|  |  |  | Guard (0.60) |
| Role_PC2 |  |  | Coworker (0.58) |
|  |  |  | Show dog (-0.68) |
| Comp_PC1 | Has your dog participated in competitions? | PCA score^P^ | Showing (0.73) |
|  |  |  | None (-0.60) |
| Comp_PC2 |  |  | Competitive obedience (0.73) |
|  |  |  | Working trials (0.81) |
| Commands | Number of commands the dog was trained for (Maximum = 10) | Numerical | n.a. |
| Train_PC1 | Which training method do you use to train your dog? | PCA score^P^ | No training (-0.85) |
|  |  |  | Motivation, positive (0.65) |
|  |  |  | Relationship based (0.61) |
| Train_PC2 |  |  | Clicker (0.67) |
|  |  |  | Counterconditioning (0.63) |
| Train_PC3 |  |  | Dominance (0.84) |
|  |  |  | Aversion (0.68) |
| F_walking | How often do you walk your dog? | Ordinal | Never |
|  |  |  | Few times a month |
|  |  |  | Few times a week |
|  |  |  | Daily |
|  |  |  | Multiple times a day |
| F_interaction_humans | How often does your dog interact with humans? | Ordinal | Never |
|  |  |  | Few times a month |
|  |  |  | Few times a week |
|  |  |  | Daily |
|  |  |  | Multiple times a day |
| F_interaction_dogs | How often does your dog interact with other dogs not living in the same household? | Ordinal | Never |
|  |  |  | Few times a month |
|  |  |  | Few times a week |
|  |  |  | Daily |
|  |  |  | Multiple times a day |
| F_training | How often do you train your dog? | Ordinal | Never |
|  |  |  | Few times a month |
|  |  |  | Few times a week |
|  |  |  | Daily |
|  |  |  | Multiple times a day |
| F_exercise | What is the amount of daily exercise your dog gets? | Ordinal | < 1h |
|  |  |  | 1 - 2h |
|  |  |  | 2 - 4h |
|  |  |  | > 4h |
| F_offlead | How often is your dog off lead? | Ordinal | Never |
|  |  |  | Few times a month |
|  |  |  | Few times a week |
|  |  |  | Daily |
|  |  |  | Multiple times a day |

n.a. = not applicable

^C^Black and tan, black and red, black and silver

^P^For multiple choice questions, a PCA was applied to condense the answers. Loadings of answer choices that contributed most to the PC are given in parentheses

*Models*

|  | Stranger-directed aggression | Dog-directed aggression | Stranger-directed fear | Human-directed playfulness | Excitability | Separation anxiety | Lack of obedience | Stranger-directed interest | Attachment/ Attention seeking | Chasing | Non-social fear | Dog-directed fear | Touch-sensitivity |
| --- | --- | --- | --- | --- | --- | --- | --- | --- | --- | --- | --- | --- | --- |
| Population (UK or Sweden) | **x** | **x** |  | **x** |  |  | **x** | **x** | **x** | **x** |  | **x** |  |
| Number of people living in the household |  |  |  |  |  |  | **x** |  |  |  |  |  |  |
| Number of children ( < 15 years) living in the household |  |  |  |  |  |  |  |  |  |  |  |  | **x** |
| Number of dogs living in the household |  | **x** |  |  |  | **x** | **x** |  | **x** |  |  | **x** | **x** |
| Do other animals live in the household (yes/ no) |  |  | **x** |  |  |  |  | **x** |  | **x** |  |  |  |
| Living place |  |  |  | **x** | **x** |  |  |  |  |  |  |  |  |
| Age | **x** | **x** | **x** | **x** |  |  |  | **x** |  | **x** | **x** | **x** |  |
| Gender |  |  |  |  |  |  |  |  |  |  |  |  |  |
| Neuter status |  |  |  |  |  |  |  |  |  |  |  |  |  |
| Gender*Neuter status |  |  | **x** | **x** |  | **x** |  |  | **x** |  | **x** | **x** |  |
| Coat colour |  |  |  |  |  |  |  |  |  | **x** |  |  |  |
| Age.acquisation |  |  |  | **x** |  |  |  | **x** |  |  |  | **x** | **x** |
| Bred |  |  | **x** |  |  |  |  |  |  | **x** |  |  |  |
| Shape | **x** |  |  |  |  |  |  |  |  | **x** |  | **x** |  |
| Role_PC1 | **x** |  |  |  |  | **x** |  |  |  |  |  |  |  |
| Role_PC2 |  |  |  | **x** |  |  |  |  |  |  | **x** |  |  |
| Comp_PC1 | **x** |  |  |  |  |  |  |  |  |  |  |  | **x** |
| Comp_PC2 | **x** |  |  | **x** |  |  | **x** |  |  |  | **x** |  |  |
| Commands |  | **x** |  | **x** |  |  | **x** |  | **x** | **x** | **x** |  | **x** |
| Train_PC1 |  |  |  |  |  |  |  |  |  |  |  |  | **x** |
| Train_PC2 |  |  |  |  |  |  |  |  |  |  |  |  | **x** |
| Train_PC3 |  | **x** | **x** |  |  |  |  | **x** |  |  |  |  |  |
| F_walking | **x** |  | **x** |  |  | **x** |  |  |  | **x** |  |  |  |
| F_interaction_humans | **x** |  |  |  |  |  |  | **x** |  |  |  |  |  |
| F_interaction_dogs |  | **x** |  | **x** |  |  |  |  |  |  |  | **x** | **x** |
| F_training |  |  |  |  |  |  | **x** | **x** |  | **x** |  |  |  |
| F_exercise |  |  |  |  |  | **x** |  |  | **x** |  |  |  |  |
| F_offlead | **x** |  |  |  | **x** | **x** | **x** |  | **x** | **x** |  |  |  |

**S3 Figure.** Principal component analysis of the genomic data. Eigenvalues for the first two principal components are plotted and individuals are coloured according to their cohort (blue=UK or pink=Sweden).


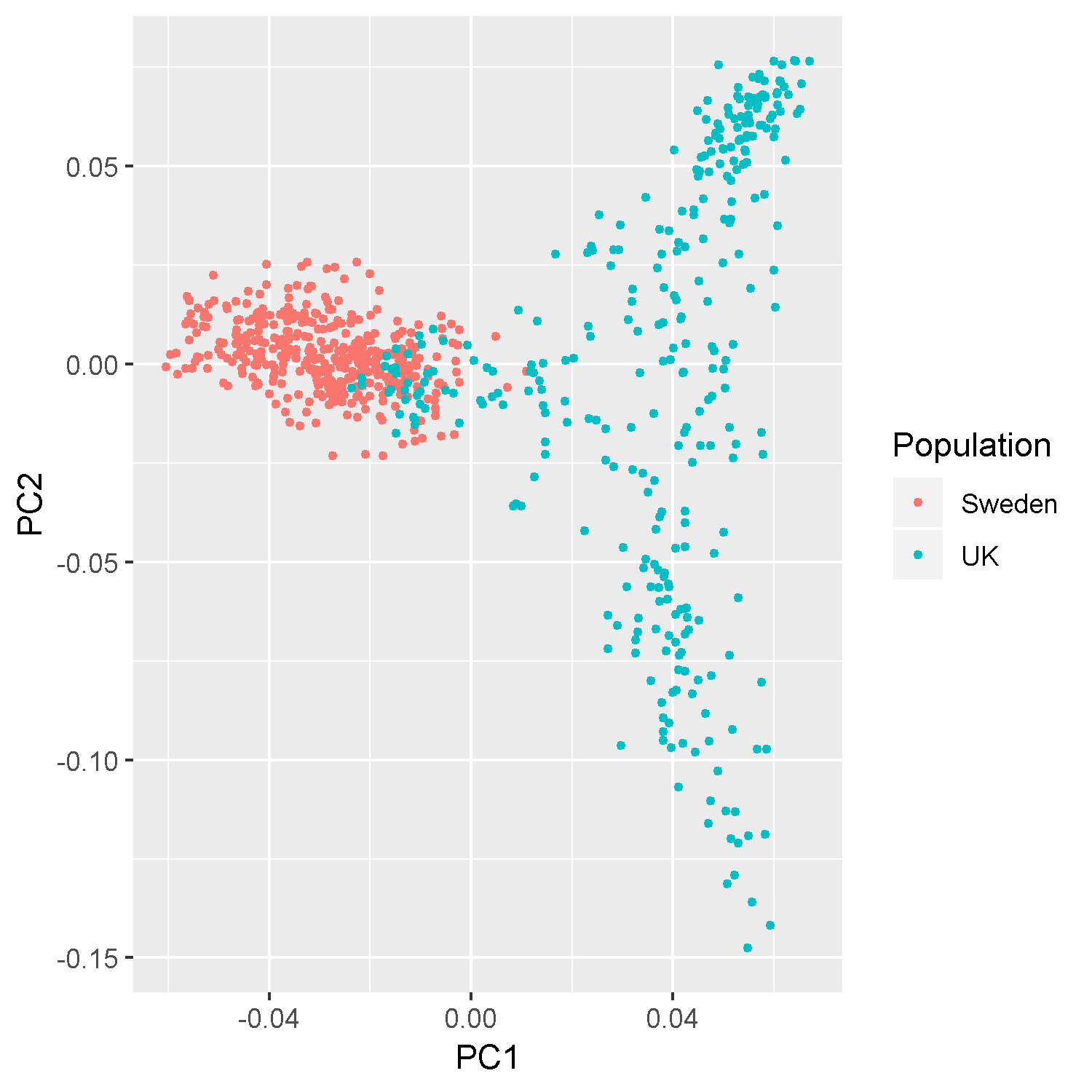


**S4 Figure.** Q-Q plots and lambda values in parentheses for the genome-wide association study of the 13 behaviour traits.


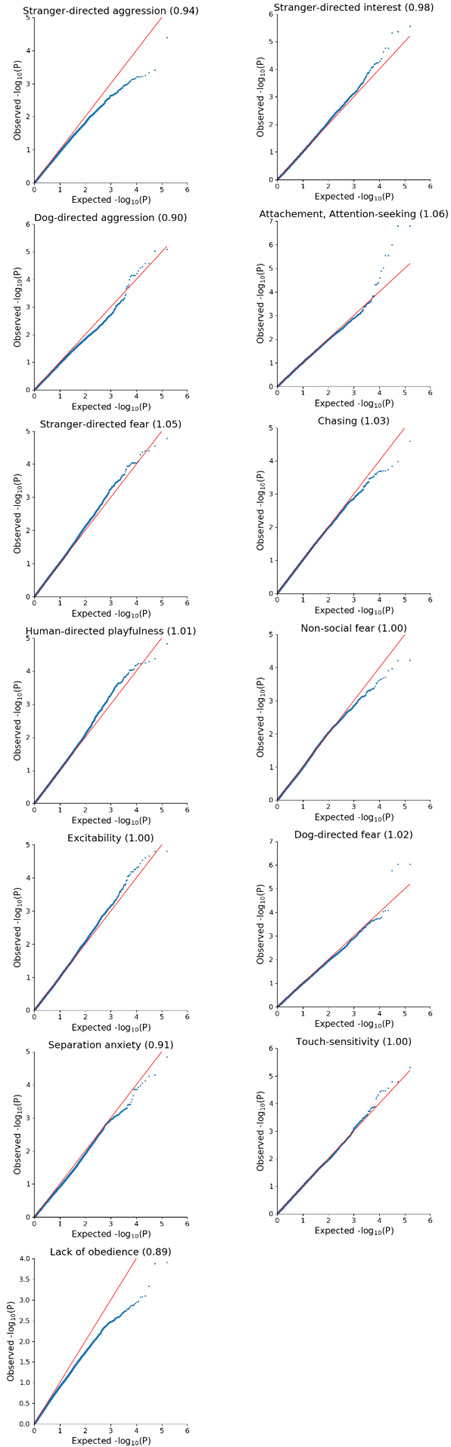


**S5 Figure**. Regional association plot. The -log(P) values calculated in the GWAS, gene annotations and local linkage disequilibrium patterns are plotted for regions identified by the regional heritability mapping that harbour genes. Neighbouring and overlapping regions (due to the sliding-window approach) were plotted together. The SNP with highest -log(P) from the GWAS is coloured in blue and all others are coloured according to their r^2^ to this SNP with white for no LD (r^2^≤0.2), yellow for weak LD (0.2≤r^2^<0.5), orange for moderate LD (0.5≤r^2^<0.8) and red for strong LD (r^2^≥0.8).
